# Online Behavioral Screener with Tailored Obesity Prevention Messages: Application to a Pediatric Clinical Setting

**DOI:** 10.3390/nu13010223

**Published:** 2021-01-14

**Authors:** Sarah Chau, Samantha Oldman, Sharon R. Smith, Carolyn A. Lin, Saba Ali, Valerie B. Duffy

**Affiliations:** 1 Department of Allied Health Sciences, University of CT, Storrs, CT 06269-1101, USA; sarahchau16@gmail.com (S.C.); oldman415@gmail.com (S.O.); saba.ali@uconn.edu (S.A.); 2 CT Children’s Medical Center, University of CT School of Medicine, Hartford, CT 06269-1101, USA; Srsmith@connecticutchildrens.org; 3 Communications Department, University of CT, Storrs, CT 06269-1101, USA; carolyn.lin@uconn.edu

**Keywords:** diet, physical activity, pediatric obesity prevention, eHealth, message tailoring

## Abstract

Obesity prevention involves promoting healthy eating and physical activity across all children. Can we leverage technology to feasibly survey children’s health behaviors and deliver theory-based and user-tailored messages for brief clinical encounters? We assessed the acceptability and utility of an online pediatric-adapted liking survey (PALS) and tailored messages among children receiving non-urgent care in a pediatric emergency department (PED). Two hundred and forty-five children (average age = 10 years, racially/ethnically diverse, 34% overweight/obese from measured indices, 25% of families reporting food insecurity) and their parents/caregivers participated. Each reported the child’s activity and behaviors using the online PALS and received two to three messages tailored to the responses (aligned with elaboration likelihood and transtheoretical models) to motivate behavioral improvements or reinforce healthy behaviors. Most children and parents (>90%) agreed the PALS was easy to complete, encouraging thought about their own/child’s behaviors. The child’s PALS responses appeared reasonable (fair-to-good child–parent intraclass correlations). Most children and parents (≥75%) reported the tailored messages to be helpful and favorable for improving or maintaining the targeted behavior. Neither message type (motivating/reinforcing) nor favorability responses varied significantly by the child’s weight or family’s food security status. In summary, children and parents found the PALS with tailored messages acceptable and useful. The message types and responses could help focus brief clinical encounters.

## 1. Introduction

Worldwide, 38.5% of children are overweight or have obesity [1]. The US 2015–2016 National Health and Nutrition Examination Survey (NHANES) revealed an 18.5% prevalence of obesity in children [2], with the highest rates among Hispanic and non-Hispanic black children [3]. Unhealthy diets, excessive screen time, and inadequate moderate-to-vigorous physical activity are well-established obesity risk factors. Lack of access to healthy food or household food insecurity contribute to unhealthy diets [4]. The prevention of childhood obesity can involve primary, secondary, and tertiary approaches to promote healthy behaviors in all children and those with the highest risk of developing obesity, as well as children with obesity [5].

Tailoring health information through a computer interface to record, process, and deliver unique information has produced small but consistent improvements in diet and physical activity behaviors [6]. Consistent with the elaboration likelihood model of attitude change [7,8], tailoring information increases its relevance and encourages individuals to think about, remember, and act on this information. Clinically based programs, especially for younger children, are usually tailored to parents’ behaviors [9] and/or parent-reported child behaviors [10]. For example, one program involved parent interaction with a kiosk, which generated a tailored child safety message from the parent’s reported knowledge and beliefs [9]. For obesity prevention, preliminary evidence supports the feasibility of tailoring to information collected via pre-visit parent telephone calls [10]. It is unknown whether tailoring messages to parents provides enough engagement for children in the behavior change processes, or is a feasible prevention/intervention approach during clinical encounters.

Effective tailored messages are usually segmented and customized to target audiences and are user customized [11], including alignment with stages of readiness to change. Readiness to change can be assessed via the transtheoretical model (TTM) [12], with interventions that encourage contemplation and preparation for behavior change, action, and maintenance. For example, a TTM-based, tailored message mobile app was developed and content validated to promote healthy dietary intake in elementary children [13]. Nonetheless, an empirical gap remains in testing and validating feasible tailored message programs that engage children, involve parents, and provide practitioners with children’s behavioral intentions to capitalize on teachable real-time and point-of-service clinical encounters.

Pediatricians embrace the need for effective obesity prevention during clinical encounters to motivate healthier diet and physical activity behaviors [14]. The pediatric emergency department (PED) should be part of obesity prevention [15], by reaching income-disadvantaged children who are unconnected with primary care [16], are at-risk for unhealthy behaviors [17,18], and need accurate communication of their weight status [19]. Before the coronavirus 2019 pandemic, the PED saw increases in income-disadvantaged children who received non-urgent care [20]. Consistent with other types of prevention efforts [21] and the goal to increase equity in obesity prevention [22], the PED is a novel location to reach at-risk children without regular primary care. Furthermore, during the pandemic, technology may help reach children for obesity prevention—especially those with health disparities [23]—for referral to programs tailored to children and parents [24], as well as screening for and informing on food security resources in a manner that minimizes stigma [25].

The current study aimed to determine the acceptability and utility of an online behavioral screener—programmed with child-tailored, obesity prevention messages—in a PED. In busy pediatric settings, the screening of modifiable behaviors is feasible [26] to guide brief, teachable encounters to promote healthy behaviors for children in all weight categories [14]. Behavioral screeners must be easy to complete and understand, in order to facilitate participation, accurate reporting, and avoid parental resistance [27]. The pediatric-adapted liking survey (PALS), a simple proxy of behaviors that correlates with biomarkers of dietary intake and indirect measures of adiposity in children [28,29], is feasible for clinical settings, taking minutes to complete, with good test–retest reliability from clinical setting to home [30]. Although children and parents interfaced with and responded to the online PALS and messages, the focus of the present study was the child. Important to the aim was to evaluate whether tailoring messages to the child’s behavioral responses was reasonable by examining the child’s behavioral reporting against that reported by the parent. The utility of the PALS and messaging program was assessed by the willingness of the child to change or maintain the message target behavior. As clinical visits must encourage child and parent participation [31] for effective obesity prevention [32], the acceptability and utility of the PALS and message program was examined in child/parent dyads. Because the program was designed to promote healthy behaviors for obesity prevention and to reach income-disadvantaged children, acceptability and utility were evaluated by the child’s weight status and proxies of family income.

## 2. Materials and Methods

### 2.1. Participants

A convenience sample of children and parents was recruited into a single session for administering the online PALS and tailored messages. The Connecticut Children’s Medical Center (CT Children’s) PED was the location to recruit children, along with their parents, who were admitted to the PED for non-urgent care in 2018–2019. The study was conducted in accordance with the Declaration of Helsinki, and the protocol was approved by CT Children’s Institutional Review Board (CCMC IRB# 13-017). Parents provided written, informed consent and completed Health Insurance Portability and Accountability (HIPAA) forms for personal health information gathered. All children assented to participate; children > 7 years provided written assent. English speaking, parent–child dyads were able to participate if children were between 5 and 17 years old, well enough to participate (healthcare provider reported, child assented), and without a history of behavioral/psychiatric illness.

### 2.2. Procedures

Research assistants approached child–parent dyads in the children’s examination room for voluntary participation. All procedures took place in this room. Enrollment times varied at the research team’s convenience. After consenting, parent–child dyads were given a touch-screen tablet computer to complete the PALS with tailored messages online via a secure Qualtrics platform. Parents responded to demographic questions (child age, race/ethnicity, gender, private/public insurance) and health-related questions (chronic medical problems; dental health—times/day brushing teeth, lifetime number of cavities, oral health rating—excellent, very good, good, fair, poor). They also were asked a 2-item family food security screener [33]; if “sometimes” or “often true” was reported for either question (i.e., worrying about running out of food and insufficient money to purchase food), parents received a private email about Supplemental Nutrition Assistance Program benefits and local emergency food centers.

The electronic medical record provided the children’s weight, height, and body mass index percentile (BMIP), categorized as underweight (<5th percentile), normal weight (5th–85th percentile), overweight (85th–95th percentile), or obese (>95th percentile) [34].

### 2.3. Intervention—Behavioral Screening and Tailored Message Program

Children and parents completed the online PALS separately, rating its acceptability and usability. From the PALS responses, they received tailored messages and rated their liking of either changing or maintaining the target behavior of the message as well as perceived message acceptability and usability.

*PALS* is a behavioral screener, construct/criterion measurement validated with 925 child–parent dyads in a paper/pencil format [29] and pilot validated with 525 child–parent dyads in an online format [35]. The online PALS assessed the participant’s level of liking/disliking of 1 practice (fun park) item that was always first in addition to 32 randomized items [29,30,35]: 3 each for fruits, vegetables, dairy, protein foods, high-fiber foods, salty foods, sweet foods, sugary drinks; 3 each for screen time and physical activities; 1 for brushing teeth; and 1 repeated (French fries) for internal test–retest reliability. Ratings were made on a continuous horizontal hedonic scale (7 faces labeled “love it,” “really like it,” “like it,” “it’s okay,” “dislike it,” “really dislike it,” “hate it”). Children and parents practiced sliding the “dot” along the online scale and then independently completed the PALS. For analysis, Qualtrics provided a ±100-point continuous item scoring (±70 to ±100 “love/hate it,” ±70 to ±40 “really like/dislike it,” ±40 to ±10 “like/dislike it,” +10 to −10 “OK”).

*Tailored health messages* were developed through prior interactions with 300+ middle schoolers, refined by dietetics and communication professionals [35], and pilot tested with direct feedback from a smaller group of children [36] and then online with 525 child–parent dyads [35]. The final messages contained attention-getting words and vivid images to engage, support, and motivate behavior change instead of health risks [37]; these messages avoided the mention of dieting, restriction, or weight loss messages that can produce poor outcomes [38]. Consistent with the elaboration likelihood model of attitude change [7], messages were tailored to children’s liking/disliking responses, with pictures matched to the child’s age group (i.e., 5 to 8 years, 9 to 12 years, or ≥13 years).

Qualtrics-programmed algorithms were developed from food/activity liking distributions through pilot testing (*N* = 535 children) to deliver 2 to 3 tailored messages per child [39]. Since research on the TTM in low-income families to promote healthier diet and physical-activity behaviors in their children provides stronger support for two (pre-action, action/maintenance) rather than five TTM stages [40], the message type was consistent with the validated 2-stage TTM [41] and avoided challenges of asking children to contemplate and report on their stage of behavior change directly [42]. Children reporting less healthy liking (volitional phase of precontemplation, contemplation, preparation), within distribution criteria (e.g., ≥ “dislike” rating for vegetables, ≥ “love it” rating for sugary beverages), received messages to raise awareness and motivate healthier options. Children reporting healthier liking (motivational phase of action and maintenance) within distribution criteria (e.g., ≥ “love it” rating for fruits or physical activities), received reinforcing messages to maintain healthy behaviors. Pilot testing revealed that 84% of children received tailored messages [35]. Generic messages (improving water consumption, dental hygiene) also were delivered, when distribution criteria were unmet.

*Post-message nutrition education*. Research assistants provided each child/parent dyad a handout with “My Weight Ruler,” communicating children’s BMIP in a culturally relevant manner [43], and simple messages to promote healthy weight (e.g., consumption of water vs. sugary beverages, MyPlate fruit/vegetable recommendations, school meal participation, daily moderate-to-vigorous activity). The assistant also briefly discussed the message, which the dyad picked as most useful, with a reinforcing handout and additional information.

### 2.4. Measures

The following measures were completed online by children and parents and before the post-message nutrition education as described.

*Acceptability and utility*. The questions for acceptability and utility were based on a single question each from the constructs of the Usefulness, Satisfaction, and Ease (USE) Questionnaire [44] to allow children as well as their parents to complete these ratings in a single clinical encounter. After rating the liking/disliking of foods/beverages and activities, participants evaluated PALS acceptability (I could answer the questions quickly without help; I could fix my mistakes easily and quickly) and usability (The questions made me/my child think about what I eat and what I do), on a continuous scale shown with 7 faces with labels of ranging from “*strongly agree*” *to* “*strongly disagree*.” After receiving all messages and reporting the liking/disliking of target message behavior, participants reported message acceptability (I learned new information about food and nutrition from these messages; The messages I received were helpful) and utility (I would like to receive more messages like these in the future).

**Figure 1:**
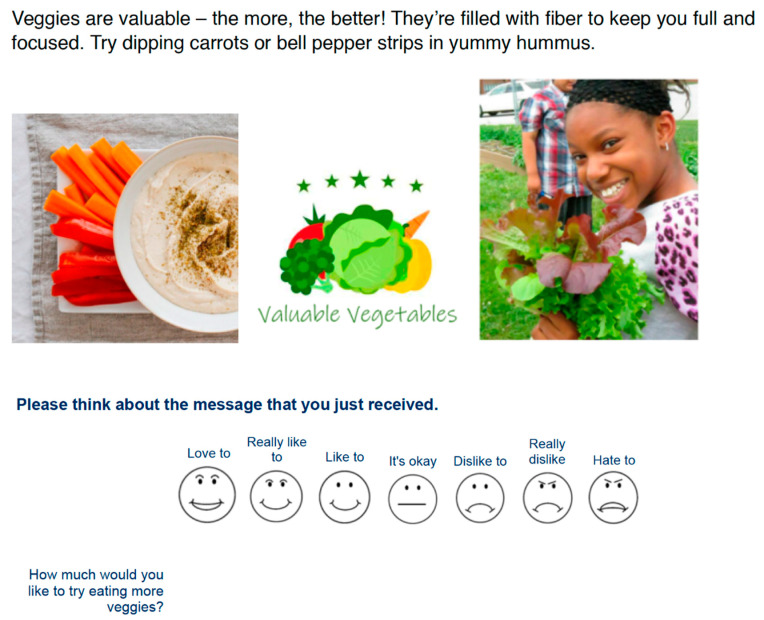
Example of a tailored message (top) to motivate a healthier behavior and the follow-up message to assess willingness to try the healthier behavior.

*Willingness to change or maintain behaviors*. After being exposed to each tailored message, participants rated how much they would like to try the behaviors suggested in messages via the labeled hedonic scale with reinforcing pictures (Figure 1).

### 2.5. Data Analysis

Data were analyzed using Microsoft Excel (version 15.13.1, Microsoft Corp., Redmond, WA, USA) and SPSS (version 25.0, SPSS Inc., Chicago, IL, USA). The significance criterion was *p* < 0.05. Descriptive statistics presented demographic and health data, PALS responses, message number, and message type, as well as usability and acceptability of PALS and tailored messages. The accuracy of children’s PALS responses was assessed against the parent-reported responses for the child and analyzed with intraclass correlation coefficients (ICC) and categories of agreement (poor 0.0 to 0.20, fair 0.21 to 0.40, moderate 0.41 to 0.60, good 0.61 to 0.80, very good 0.81 to 1.00) [45]. Paired *t*-tests computed mean differences between children and parents and provided input for visual inspection of the Bland–Altman plots to evaluate proportional bias between the two scores. Differences in PALS and message acceptance and utility by child demographic and weight status as well as family income were assessed by non-parametric statistics (χ^2^ for categorical, Spearman rho statistic for continuous variables) and analysis of covariance (ANCOVA)—controlling for demographic variables—with Levene’s test for homogeneity of variances across levels of independent variable.

## 3. Results

### 3.1. Descriptive Results

Overall, 396 dyads were approached and 72 of them declined (82% acceptance). Of the 324 dyads recruited, 79 enrollments were incomplete due to tablet or internet malfunctions, missing survey responses, patients becoming sicker, and others (e.g., medical care interruptions).

The final sample yielded 245 child/parent dyads, diverse in age, gender, and race/ethnicity, with 60% on public health insurance (Table 1). Overall, 33% of children were characterized with overweight or obesity, which is comparable to the US average of 36.6% [2]. Food insecurity was reported by 22% of parents, higher than the 12.4% reported for Connecticut [46].

### 3.2. Description of PALS Responses

**Figure 2:**
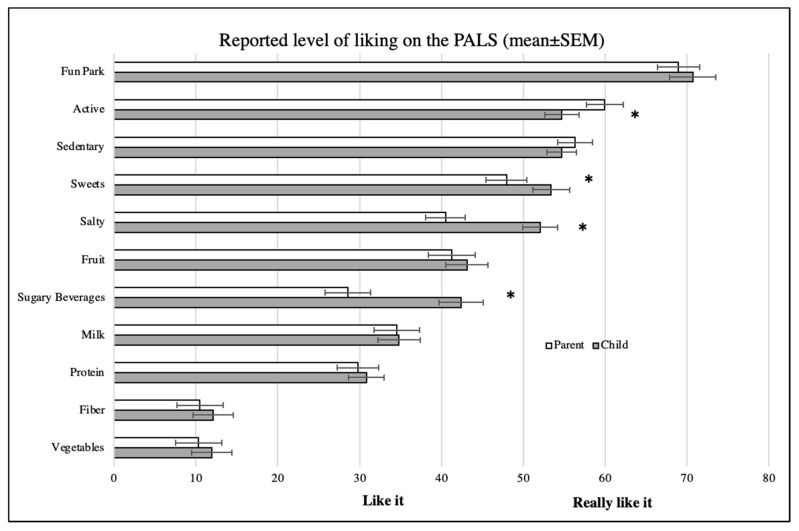
Reported level of liking on the pediatric-adapted liking survey (PALS) by parents and children (mean ± SEM). * Indicates significant mean differences (*p* < 0.05).

The sample showed good variability in food and physical activity liking/disliking (highest for activities, lowest for vegetables and high-fiber foods) (Figure 2). The single food (French fries) test–retest reliability was very good for children (r = 0.88) and excellent for parents (r = 0.90). The internal reliability of individual groups (i.e., Cronbach’s alpha) across children and parents ranged from acceptable/approaching acceptable (vegetables, fruit, sweets, sugary drinks, salty, physical activity) to below acceptable (dairy, screen time, fiber, protein).

Parent–child ICC’s ranged from 0.331 for fiber foods (fair) to 0.583 for dairy (moderate), extending to “fair” for salty and screen time and “moderate” for physical activity, protein, sweets, sugary drinks, and vegetables. In comparing average liking, parents reported their child with more favorable liking for healthier activities and foods than the children, significant for physical activity, sweet, and salty groups (Figure 2). The Bland–Altman plots generally did not show proportional bias.

The online PALS was acceptable to children and parents, with 94% and 97–98% reporting at least “somewhat agree” to answering the questions quickly without help and fixing mistakes easily, respectively. Children and parents also “agreed” the PALS made them think about what they do or what their children do (93% and 98%, respectively). In paired *t*-tests, parent/child dyads did not differ significantly in being “able to complete the survey quickly” (t = 0.89, *p* = 0.36) or the survey “made them think about what they do” (t = 0.66, *p* = 0.51). Children had significantly lower mean agreement with “ability to fix their mistakes” (when interfacing with PALS) than parents (t = 2.15, *p* < 0.5), but averaged in the “agree” range. Child age was associated significantly with greater agreement with “ability to complete the survey quickly without help” (rho = 0.17, *p* < 0.01) but lower agreement that “the survey made them think about what they do” (rho = −0.23, *p* < 0.01).

### 3.3. Tailored Health Messages

Children averaged receiving just over two tailored messages (2.14 ± 0.98 SD), and within a child, received significantly more motivational than reinforcing messages (1.24 ± 0.92 versus 0.91 ± 0.99, respectively, t = 0.3.21, *p* < 0.01). In chi-square analysis, children in the oldest age category (≥13 years) were less likely to receive reinforcing messages (χ^2^(4) = 12.98, *p* < 0.05) and boys were more likely to receive motivational messages (χ^2^(2) = 6.00, *p* < 0.05). The number of motivational or reinforcing messages did not differ significantly by insurance type, family food security, or child BMIP category.

Most motivational messages received by children entailed the consumption of fewer sweets, salty foods, and sugary drinks, and engaging in less screen time (approximately 20% for each). In contrast, approximately the same percentage of children received reinforcing messages about continued engagement in physical activity and consumption of fruits or dairy products.

### 3.4. Relevance and Acceptance of Tailored Messages

The tailored messages were generally deemed acceptable and relevant to children and parents, with a rating of at least “somewhat agree” to “learning new information” (89 versus 78%, respectively) and “messages I received were helpful” (93 versus 86%, respectively). Children and parents also “agreed” they would like to “receive more messages like these in the future” (88 and 75%, respectively). In paired *t*-tests, children reported a greater level of agreement with all three statements about the tailored messages (p < 0.05 to < 0.001).

Greater child age associated significantly with a lower level of agreement on learning new information (rho = −0.22, *p* < 0.01), helpfulness (rho = −0.19, *p* < 0.01), or wanting future messages (rho = −0.24, *p* < 0.01), with the oldest age category still averaging between “agree” and “somewhat agree.” Girls provided higher ratings than boys for all message responses (*p* < 0.05 to < 0.001). Message responses did not vary significantly by BMIP, insurance type, or food security status.

### 3.5. Willingness to Change to or Maintain Healthy Behaviors

Ratings of liking to maintain healthy behaviors was significantly higher than liking to change from less healthy to healthier behavior (60.33 ± 3.99 versus 38.17 ± 3.39, respectively, *p* < 0.001). Most children (82.9%) reported ≥”like” to changing at least one behavior from less healthy to healthier. As shown in Table 2, willingness to change behaviors in response to motivational messages (rating > ”It’s okay”) varied between 50% of children receiving messages to increase consuming fiber foods and 86% of children receiving messages to decrease consuming sugary drinks.

Older children trended toward lower liking to change to healthier behaviors (rho = −0.14, *p* = 0.054), but no difference in liking to maintain healthy behaviors (rho = 0.65, *p* = 0.442). These liking ratings did not differ between girls and boys (*p* = 0.11–0.83), by family insurance (*p* = 0.86–0.89) or food security status (*p* = 0.36–0.57). ANCOVA results showed no significant difference across BMIP categories for either average liking to change to healthier behaviors (F(2,172)) = 0.750, *p* = 0.47) or average liking to maintain healthy behaviors (F(2,131)) = 0.268, *p* = 0.77).

In comparing within dyads, parents provided significantly higher average ratings for their child’s liking to change to healthier behaviors (60.92 ± 2.66 versus 35.47 ± 4.00, t = 5.493, *p* < 0.001) and only trended toward higher average liking to maintain healthy behavior (72.47 ± 3.03 versus 64.55 ± 4.07, t = 1.67, *p* < 0.1).

## 4. Discussion

Obesity prevention should reach all children to promote enjoyable healthy eating and physical activities. eHealth offers a way to reach children and families with tailored health information, which may be increasingly important for income-disadvantaged children without access to regular healthcare [23]. The present study reached racially/ethnically diverse children (a majority from families of economic disadvantage) who were evaluated in the PED for non-urgent care. Both child and parent participants reported high acceptability and usefulness of both the PALS (behavioral screener) and tailored messages, which aimed to motivate behavioral improvement or reinforce healthy behaviors. Most children (82.6%) indicated liking or a favorable response to change toward healthier behaviors. The acceptability and usability of PALS and tailored messages varied significantly neither with children’s weight status, nor with indicators of the families’ income. Thus, this program could be a useful tool for obesity prevention in clinical settings as an initial step towards encouraging children to adopt healthier behaviors.

From previous studies, PALS was found to be a feasible and reasonable indicator of usual dietary intake and, we suspect, of usual screen time and physical activities. PALS takes <5 min for children to complete offline [29,30] and online [35]. Presently, nearly all children and parents agreed that the online PALS was easy to complete and made them think about their and their child’s behaviors, respectively. Summarizing previous justification [29], we ask liking/disliking with the basic assumption that, over time, we do what we like and avoid what we do not. There is a correlation between reported food and beverage liking/disliking as well as intake in children [28,47] and adults [48,49,50,51,52,53]. Individuals may restrain their liking of good-tasting foods for health reasons [48,51,54] and eat less-liked foods that are healthy [28,51]. Patterns of food-liking correlate with biomarkers of dietary intake and/or indirect measures of adiposity in children [28] and adults [51,55,56,57].

Within dyads, children’s liking for foods/beverages and physical activities were generally similar to parents’ reporting of their children’s behaviors. Initially, we had speculated that the parents were the truer report to compare the child against, without having a more definite assessment of behavior (e.g., multiple assessments with biomarkers of consumption or activity). Nonetheless, a number of findings support that the children’s responses were reasonable and perhaps more forthcoming indications of usual behaviors to tailor health promotion messages. Children and parents did not report a significant difference in average rating of liking for a fun park, which served to orient participants to PALS and supported that children and parents were using the scale consistently [58]. The test–retest reliability of a repeated item was very similar for children and parents (reflecting “very good” to “excellent,” respectively). Our findings with child–parent behavioral reporting are in agreement with those from previous studies—children averaged less favorable liking for sweets, salty snacks, sweet beverages, and physical activities, but non-significant differences for healthier foods [59,60]. Overall, this suggests that tailoring messages to the children’s reported behaviors may be useful, especially to focus attention on less healthy behaviors. Parents may falsely report that their child engages in healthier behaviors to reflect a more socially desirable response [61]. Similarly, parents also reported a significantly greater liking of physical activities than did the child [62]. In clinical practice, having both the child and parent report on the child’s behavior, receive the tailored messages, and discuss similarities/differences might inform brief motivational interviewing encounters to help children and parents identify shared behavioral change goals and planned actions [63].

The utility of the PALS and tailored messages was reported directly by children and parents, and indirectly, through reported willingness (liking) to try healthier behaviors. The observed level of acceptability is similar to that reported for tailored communications for obesity prevention in primary care [10]. The PALS prompted children to reflect on their behaviors, an important initial step to behavior change. The messages were TTM aligned, which supports behavior changes in children [6] via motivating improvements for less healthy behaviors and reinforcing healthier behaviors. Messages motivating behavioral improvements were positive and non-judgmental, with suggestions of alternative and healthier options.

In clinical settings, including the PED, willingness to try healthy behaviors can focus patient–provider communications for practitioners [26] on brief motivational interview sessions between the practitioner and child/parent dyad. These sessions typically involve reflective listening, autonomy support, shared decision making, and change talk [64] to increase motivation for behavior change, including among diverse adolescents [65]. The present study included a reinforcing handout on the tailored message and communicating weight status with culturally acceptable methods [43], which can encourage appropriate behavior change goals that arise from practitioner, child, and parent interactions [66]. Children’s responses to behavior change support personal choice and, for older children, align with the self-determination theory, which asserts the role of intrinsic and extrinsic motivation in behavior choice [67]. Involving parents in the dialogue can promote: joint child–parent problem solving to make healthier options available; food choices within “liked” healthier options; more positive support from parents; and parents modeling healthy behaviors as role models, including through family meals [68]. For example, lower consumption of sugary drinks is supported with children’s willingness, coupled with low access in the home, and the supportive attitudes and behaviors of parents [69].

The present study has strengths and limitations. The PALS is validated for use in clinical settings [29], with responses as reasonable proxies to those at home [30]. In particular, it can assess an individual’s usual behaviors and tailor the messages as feedback within the framework of behavior change theories. The PALS, a brief but thorough behavioral screener, is easy for children and parents to use, successfully prompting willingness to complete, and minimizing barriers associated with low adherence [27]. Our work with middle schoolers [70] supports that additional instructions are not required beyond what is embedded in the online survey. This online platform is interactive via pop-up health promotion messages tailored to children’s survey responses, without demands on the practitioner’s time to analyze, as found previously [27]. The PALS results and/or tailored messages are sharable, with follow-up messages to the parent [35].

As a limitation, the study was conducted only with English speakers. While we have shown that liking ratings in the PED and at home show good/excellent test–retest reliability, the acceptability and utility responses of the messages should also be tested beyond the PED and into the home, with follow-ups in primary care as well as assessment of the impact on behavioral change and weight outcomes (e.g., [71]). Nonetheless, the study was unique in that messages were tailored to children’s responses. Although not tested, the PALS and tailored messages could be completed pre-visit and the results could be emailed to the physician for focused attention during the pediatric visit. Likewise, the PALS and the message program could be administered via a smartphone, with results emailed to the practitioner [72]. Although our previous study showed that completing the PALS by itself was feasible in a clinical setting [29,30,35], the present study and online platform did not differentiate the time of completion of the PALS from that of responding to the messages, the message evaluation, and user acceptability ratings as well as activities that may not be needed in clinical practice (e.g., time required for the consenting process, metadata collection). This study also showed that family food insecurity can be discreetly assessed via online screening—followed by emailing community resources to families directly—to minimize stigma [25] and support improved food access for obesity prevention and health [73].

Presently, neither practitioner acceptability of the PALS nor the messaging program to practitioners was tested. Rather, we tested acceptability and usefulness to children and parents, including the children’s willingness to try healthier behavior options. The sample, although diverse and income disadvantaged, was of convenience and lacked a randomized control group design for comparison. The impact of the PALS and the messaging program on behavior changes is not totally known. Change in actual behaviors requires a process with children and parents engaged in goal setting, monitoring, positive reinforcement, stimulus control, modeling, problem solving, and preplanning. Nonetheless, the PALS administered in combination with a single session of tailored messages could start the behavior change process and identify the behaviors the child might most likely change with the support of their parents.

## 5. Conclusions

This feasibility study supported that the online PALS with tailored messages delivered in a clinical setting was acceptable and useful to children and parents. The comparison between child reporting and parent reporting of their child’s behavior supports that children were reasonable enough in reporting their usual dietary and physical activity behaviors—to receive tailored health promotion messages—via interfacing with the PALS online. The online platform of the PALS and message program could broaden the reach for obesity prevention, especially for those without regular or sufficient healthcare access. The screener and message program did not require skilled technicians for interpretation and forming recommendations. Future testing will determine practitioner acceptance of the PALS and the message program and effective clinical integration, and the potential for incorporation into multi-component obesity prevention efforts.

## Figures and Tables

**Table 1:** Characteristics of children seeking medical care in a pediatric emergency department (PED).

	*N* = 245	%
**Age (Average = 10.2 Years)**		
5 to <9 years	88	36
9 to <13 years	85	35
13 to 17 years	72	29
**Gender**		
Male	132	54
Female	110	45
Other	3	1
**Race/Ethnicity**		
White	86	35
Black	47	19
Hispanic	102	42
Asian	8	3
Other	2	1
**Insurance**		
Private	99	40
Public	146	60
**Food Security ^†^**		
Secure	184	75
Insecure	61	25
**BMI percentile ^††^**		
Underweight	15	6
Normal	148	60
Overweight	33	14
Obese	48	20

^†^ Reporting sometimes or often to either of the two food security questions. ^††^ Where underweight is determined as less than 5th BMI percentile.

**Table 2:** Health messages by topic and child’s willingness to try new health behavior.

Motivating Tailored Health Message	Willingness to Change Behavior Question	Child % Reporting “*Like*”–“*Love to*”
Sugary Drinks: Tame your thirst with water—fruit juices and sugary drinks will just make you thirstier!	How much would you like to drink more water?	86
Sweets: Cookies and candy have extra sugar that your body doesn’t like. Instead, try eating your favorite fruits as a sweet treat!	How much would you like to eat fruit as a snack or for dessert?	80
Screen time: TV and video games are fun to play, but try to limit them to 2 h a day! Instead, dance with friends, play fetch with your pup, or go to the playground with your parents! Aim to be active for 1 h each day.	How much would you like to be moving and playing for 1 h or more each day?	80
Dairy: Dairy is delightful! Try choosing yogurt, milk, and cheese. These foods deliver calcium to your body to keep your bones strong and your smile bright.	How much would you like to eat more yogurt, milk and cheese?	75
Salty Snacks: Slow down with salt! Snacks like chips and French fries are oh-so-salty. Eating them will make you thirsty. Try choosing a smart snack, like celery sticks topped with peanut butter and whole grain cereal!	How much would you like eating healthier snacks?	74
Fruit: Feast on fruit! They pack vitamins that make your skin glow and hair shine. Try topping your cereal or yogurt with a blend of berries!	How much would you like to try eating fruit at most meals and snacks?	73
Vegetables: Veggies are valuable—the more, the better! They’re filled with fiber to keep you full and focused. Try dipping carrots or bell pepper strips in yummy hummus.	How much would you like to try eating more veggies?	56
Fiber: Go with the whole grain! Try munching on whole wheat breads for your lunchtime sandwiches and brown rice when building your dinner plate!	How much would you like to eat more whole grains?	50

## Data Availability

The data presented in this study are available on request from the corresponding author.
